# Improving the routine analysis of siderite for δ^13^C and δ^18^O in environmental change research

**DOI:** 10.1002/rcm.9456

**Published:** 2023-01-16

**Authors:** Jack H. Lacey, Hilary J. Sloane, Melanie J. Leng, Stephen F. Crowley

**Affiliations:** ^1^ National Environmental Isotope Facility, British Geological Survey Nottingham UK; ^2^ Centre for Environmental Geochemistry, School of Biosciences, Sutton Bonington Campus University of Nottingham Loughborough UK; ^3^ School of Environmental Sciences University of Liverpool Liverpool UK

## Abstract

**Rationale:**

The carbon (δ^13^C) and oxygen (δ^18^O) isotope composition of siderite (FeCO_3_) is used widely to understand and quantify geochemical processes to reconstruct past climate and environmental change. However, few laboratories follow precisely the same protocol for the preparation and analysis of siderite‐bearing materials, which combined with the absence of international reference materials and mineral‐specific acid fractionation factors, leads potentially to significant differences in isotope data generated by individual laboratories. Here we examine procedures for the isotope analysis of siderite and discuss factors potentially contributing to inconsistencies in sample measurement data.

**Methods:**

Isotope analysis of siderite is first assessed using similar versions of the classical off‐line, sealed vessel acid digestion method by comparing data sets obtained from intercomparison materials measured at two participating laboratories. We then compare data from the classical method against those generated using an automated preparation technique using data produced from an independent set of test materials.

**Results:**

Measurement of siderite δ^13^C is generally both repeatable and reproducible, but measurement of δ^18^O may be subject to large (~1‰), method‐dependent bias for siderite reacted at differing temperatures (70°C and 100°C) under classical and automated CO_2_ preparation conditions. The potential for poor oxygen isotope measurement reproducibility is amplified by local differences in sample preparation protocols and procedures used to calibrate measurement data to international reference scales.

**Conclusions:**

We offer suggestions for improving the repeatability and reproducibility of δ^13^C and δ^18^O analysis on siderite. The challenge of producing consistent isotope data from siderite can only be resolved by ensuring the availability of siderite reference materials to facilitate identical treatment as a basis for minimising method‐dependent contributions to data inconsistency between laboratories.

## 1 INTRODUCTION

The stable isotope composition of carbonate sediments and fossils provides one of the most important geochemical proxies for environmental change research,^1^, ^2^ and has been used as an indicator of palaeoclimate since the seminal work of McCrea.^3^ Whilst the mineral used in the majority of studies is calcite (CaCO_3_), many carbonate‐bearing sediments do not contain calcite (endogenic or authigenic) but instead preserve other carbonates, including siderite. Siderite is a naturally occurring iron‐rich carbonate mineral (FeCO_3_) found in freshwater and marine sediments,^4^, ^5^, ^6^, ^7^, ^8^ hydrothermal and metasomatic iron mineralisation,^9^, ^10^ and carbonatite magmatism.^11^ In addition, siderite may be an important mineral on Mars, where it is implicated in the decarbonisation of the Martian atmosphere.^12^, ^13^, ^14^

Siderite can form during early diagenesis from ferrous iron [Fe]^2+^ and carbonate [CO_3_]^2−^ ions dissolved in marine, brackish, and fresh porewaters in both marine and terrestrial environments.^15^ Dissolved [Fe]^2+^ only accumulates to significant levels in anoxic waters, under the absence of O_2_ where oxidisation to [Fe]^3+^ is arrested, and with negligible sulphate concentrations to prevent the preferential formation of hydrogen sulphide and subsequent precipitation of iron sulphide.^16^ Siderite is therefore precipitated typically under strongly reducing conditions with elevated CO_2_ partial pressure, potentially accompanied by methane formation, and is generated more readily in non‐marine sediments due to the lower sulphate and higher organic matter concentrations of freshwater systems,^17^ for example due to stratified water columns in biologically productive lacustrine settings.^7^ As for calcite, the stable isotope composition of siderite can be a sensitive recorder of information about the environment of its formation, given the oxygen isotope composition (δ^18^O) of siderite is dependent on the temperature and δ^18^O of the water from which the mineral precipitated.^18^, ^19^, ^20^ Based on this relationship, siderites have been used as palaeoclimate and palaeoenvironmental indicators.^8^, ^21^, ^22^, ^23^, ^24^, ^25^

Many laboratories measure the carbon isotope composition (δ^13^C) and δ^18^O of siderite, but methods are generally laboratory‐specific, with preparation and analyses carried out under varying experimental conditions. The classical method for determination of δ^13^C and δ^18^O of carbonate minerals developed originally by McCrea^3^ is based on the off‐line preparation of CO_2_ by reaction with hyperconcentrated (anhydrous) phosphoric acid (H_3_PO_4_ ≥ 100%) in sealed vessels and analysis using isotope ratio mass spectrometry. A simplified description of reaction chemistry is summarised in equations 1 and 2.^26^, ^27^

(1) $${\mathrm{MCO}}_{3}+H_{3}{\mathrm{PO}}_{4}\to H_{2}{\mathrm{CO}}_{3}+{\text{MHPO}}_{4}$$

(2) $$H_{2}{\mathrm{CO}}_{3}\to {\mathrm{CO}}_{2}+H_{2}O$$

where M is the divalent cation.

In the phosphoric acid reaction, only two of the available carbonate oxygen atoms are transferred to product CO_2_ for isotope analysis. The partitioning and equilibration of oxygen isotopes between CO_2_, H_2_O, and a diverse range of potential oxygen‐bearing phosphate species is accompanied by a mineral‐specific and temperature‐dependent kinetic isotope effect that is corrected for by applying an appropriate, empirically derived, “acid fractionation factor” *α* ^28^, ^29^, ^30^, ^31^:

(3) $$\alpha_{{\mathrm{CO}}_{2}-\text{carbonate}}=\left(\delta^{18}O_{{\mathrm{CO}}_{2}}+1000\right)/\left(\delta^{18}O_{\text{carbonate}}+1000\right)$$

Siderite is a rhombohedral (calcite‐type structure) carbonate mineral and as iron occurs as [Fe]^2+^ there is a broad array of potential isomorphous substitutions with cations of similar radii and identical charge (e.g. Mg, Mn, Zn, Ca), therefore the acid fractionation factor will vary depending on the carbonate mineralogy. The acid fraction factor commonly used for natural siderites is 1.00881 for reactions at 100°C,^29^ but specific acid fractionation factors for different analytical methods and reaction temperatures have also been derived empirically.^18^, ^32^, ^33^

Over the past three decades there has been a general trend towards replacing the classical off‐line method with automated on‐line gas preparation procedures to increase sample throughput. To accommodate automation, extensive modifications of the original method have been adopted, with the result that few laboratories follow the same analytical protocol. There are between‐laboratory differences in (1) sample preparation (particle size, organic contaminant removal, carbonate purity, sample weight), (2) reaction vessel design (sealed versus open vessel, individual vessel versus common acid bath, static versus stirred), (3) concentration and volume of phosphoric acid, reaction temperature, and duration, (4) manual versus automated gas preparation and gas handling, (5) CO_2_ purification (cryogenic, chromatographic, membrane separation) methods, (6) gas transfer (indirect/off‐line versus direct/on‐line) to the mass spectrometer, and (7) mass spectrometry (dual inlet versus continuous flow). To minimise potential measurement differences caused by these differing analytical procedures, individual laboratories rely on the application of the Principle of Identical Treatment to mitigate local, method‐dependent effects. The principle is based on the contiguous preparation of CO_2_ from primary (international) or secondary (laboratory) carbonate reference materials and samples of unknown isotope composition under identical reaction, gas processing, and mass spectrometric conditions. Any local effects are assumed to cancel as long as the response of calibration and sample materials is effectively identical.^34^, ^35^

The application of identical treatment has proven to be a successful strategy for the routine determination of the isotope composition of samples of calcite because international reference materials (e.g. IAEA‐603, USGS44) are freely available and specimens of calcite, suitable to act as reliable secondary laboratory calibration materials (e.g. Carrara marble, calcite carbonatite, synthetic CaCO_3_), can easily be sourced. Consequently, any modification of the classical method used to measure the isotope composition of calcite can be tested and validated. Furthermore, analytical data can be normalised to the Vienna Pee Dee Belemnite (VPDB) scale using a two‐point calibration procedure that is traceable via local measurement of primary calcite reference materials.^31^, ^36^ By contrast, the absence of internationally accepted and mineral‐specific reference materials for other types of carbonate prohibits the effective application of identical treatment. For siderite, different laboratories modify CO_2_ preparation procedures (finer particle sizes, higher reaction temperatures) to reduce normally lengthy reaction times.^18^, ^29^ In addition, values of $\alpha_{{\mathrm{CO}}_{2}-\text{carbonate}}$ for calcite and siderite differ. To circumvent the fractionation problem, laboratories operating classical and automated acid digestion procedures may adopt different calibration strategies. Laboratories following classical methods may choose to react calcite calibration materials at 25°C for standard time intervals while reacting samples of siderite at much higher temperatures (≥70°C) for extended time periods. This procedure is similar to methods originally used to derive both the value and temperature dependence of commonly applied siderite acid fractionation factors^18^, ^29^ and conditions used to determine the isotope composition of primary reference calcites.^30^, ^37^ Alternatively, automated procedures (using a commercial preparation device connected to an isotope ratio mass spectrometer) typically react siderite samples and calcite calibration materials at the same temperature (usually ≥70°C), rely on published siderite and calcite temperature‐dependent oxygen isotope fractionation relationships, and assume these to be internally consistent for any given set of gas preparation conditions. Automated on‐line preparation devices may use sealed vessels for acid digestion, similar to the classical off‐line method but generally requiring less sample material, or open vessel techniques, for example where liberated CO_2_ is frozen continuously into a separate trap using liquid N_2_.

Here we examine potential method‐dependent contributions to uncertainty in the analysis of siderite by (1) assessing analytical repeatability and reproducibility by determining the δ^13^C and δ^18^O of four siderite intercomparison materials using similar versions of the classical off‐line acid digestion method operating in two well‐established laboratories at the British Geological Survey (BGS) and the University of Liverpool (UoL), (2) investigating key aspects of the CO_2_ preparation method, including the apparent rate of siderite reaction with anhydrous phosphoric acid, and the effects of acid concentration and reaction progress on the isotope composition of CO_2_, and (3) comparing the classical method (sealed vessel/off‐line) against two alternative procedures (open vessel/off‐line and sealed vessel/on‐line) using four test siderites originally analysed at the Geological Institute of the Eidgenössische Technische Hochschule (ETH), Switzerland.^32^, ^33^ On the basis of our results we discuss potential sources of measurement inconsistency and offer recommendations to improve the routine isotope analysis of siderite.

## 2 EXPERIMENTAL

### 2.1 Geochemical characterisation of intercomparison materials

Four siderite‐bearing intercomparison materials were prepared from natural rock samples at the BGS (Table 1 and Appendix S1). The samples were hand crushed using an agate mortar and pestle, and the resultant powders were stored in screw‐cap sealed glass vials prior to analysis. An aliquot of each siderite was further ground and split into 10–63 μm and <10 μm grain size fractions by milling with agate spheres and then micronizing, respectively, to determine the major elemental composition and mineralogy of each intercomparison material using X‐ray diffraction (XRD) and X‐ray fluorescence (XRF) at the Department of Geology, University of Leicester. For XRF, lithium tetraborate fused glass discs were prepared from the 10–63 μm powders and analysed using a PANanalytical Axios Advanced XRF spectrometer. The instrument was calibrated by measuring an appropriate range of international rock reference materials and pure oxides (Fe_2_O_3_). Loss‐on‐ignition values were determined by heating sample powders at 950°C for 1.5 h prior to the preparation of glass discs. For XRD, the <10 μm powders were analysed using a Bruker D8 Advance diffractometer equipped with a LynxEye linear position sensitive detector and using CuKα radiation over the scan range 4–90°2*θ*. The step size was 0.015°2*θ* with 0.8 s per step. Phase identification was performed using Bruker DIFFRACplus EVA search/match software interfaced with the PDF‐4+ database from the International Centre for Diffraction Data. A further four test siderites, prepared originally at ETH, were used in this study and the details of the analytical methods used to characterise these materials are reported in Fernandez et al.^33^ The chemical composition of ETH siderite test materials was determined by inductively coupled plasma mass spectrometry (ICP‐MS) analysis (Table 2).^33^ No procedural description of the analytical method is available.

**TABLE 1:** Geological context and source location of BGS siderite intercomparison materials

Material	Geological context	Source location
MR15423	Spherulitic siderite in mudstone	Leeds, Yorkshire, UK
MR17327	Siderite‐fluorite, hydrothermally altered quartz arenite	Leeds, Yorkshire, UK
MR31556	Siderite‐quartz hydrothermal mineralization	Luckett, Cornwall, UK
IVIGTUT	Siderite‐cryolite‐quartz hydrothermal mineralization	Ivigtut, Greenland

**TABLE 2:** Chemical composition of ETH siderite test materials (Alvaro Fernandez, personal communication)

Material	FeCO_3_	MnCO_3_	MgCO_3_	CaCO_3_
ES	72.074	3.323	12.790	11.812
Four	67.166	3.153	18.250	11.431
IG	83.277	3.848	1.319	11.556
LT	75.837	4.033	6.328	13.803

Values in 100 × carbonate.

Quantitative measurement of the total carbon (TC) composition of intercomparison materials was undertaken using a Thermo Scientific FlashSmart CN elemental analyser at UoL. Samples (approximately 5 mg) were weighed precisely (0.0001 mg) into tin capsules and combusted in oxygen at 950°C. Product gases were passed through an oxidation column containing copper(II) oxide and platinised alumina, and a reduction column containing copper metal wires (at 750°C). Any water vapour was removed by reaction with magnesium perchlorate. Purified CO_2_ was finally separated by chromatography and measured using a thermal conductivity detector. The instrument was calibrated using an organic sediment standard material and validated for carbonate mineral analysis by replicate (*n* = 4) analysis of spectrographically pure CaCO_3_, NBS1b limestone and NBS88a dolomite.

### 2.2 Preparation of anhydrous phosphoric acid

The partial pressure of CO_2_ and water vapour, and their residence time at elevated reaction temperatures (>> 25°C), together with the distribution of phosphate species, are potentially critical variables contributing to the specific isotope composition of CO_2_ recovered for mass spectrometric measurement.^38^, ^39^, ^40^, ^41^, ^42^, ^43^ The water content of the system includes both H_2_O present within reactant phosphoric acid and H_2_O generated as a by‐product of acid‐carbonate reaction. The quantity of “free” water in phosphoric acid (and the distribution of phosphate species) is sensitive to the polymerisation of phosphoric acid (e.g. 2H_3_PO_4_ ↔ H_4_P_2_O_7_ + H_2_O) as the P_2_O_5_ content increases above 69 wt%.^44^

Anhydrous phosphoric acid is typically produced by one of five methods^40^: (1) addition of P_4_O_10_ to stock 15 M (85% H_3_PO_4_) orthophosphoric acid,^45^ (2) vacuum distillation or (3) boiling of stock orthophosphoric acid, (4) melting of crystalline H_3_PO_4_ ^46^ or (5) addition of H_2_O to P_4_O_10._ ^41^ The procedure by which anhydrous phosphoric acid is synthesised may result in the production of acids of differing concentration, proportion of orthophosphoric, di‐phosphoric and tri‐phosphoric acids, distribution of polymer chain lengths, and quantity of cyclic (metaphosphoric) derivatives.^44^, ^47^, ^48^ The addition of H_2_O_2_ and CrO_3_, as recommended in early acid preparation protocols,^40^, ^45^ further increases the range of variables contributing to differences in acid chemistry and oxygen isotope composition. No systematic studies of the potential isotope effects of differing anhydrous phosphoric acid preparation procedures are available. It is normally assumed that the isotope composition of CO_2_ released by the carbonate‐acid reaction is independent of the method of manufacture, but it is evident that both the physical and chemical properties of anhydrous phosphoric acid can vary. For example, production by vacuum distillation is thought to minimise the occurrence of cyclic derivatives that may occur in acid prepared by P_4_O_10_ addition.^47^

At BGS, acid preparation follows the procedure described by Coplen et al.^45^ in which approximately 2000 g of P_4_O_10_ is added to 2.5 L of analytical grade 15 M H_3_PO_4_ heated to a temperature of 160°C. CrO_3_ and H_2_O_2_ are added to ensure complete removal of organic contaminants. The notional concentration of H_3_PO_4_ is determined by titration against 0.1 M NaOH using bromocresol green indicator and a hydrometer. This procedure results in a final product with a specific gravity of approximately 1.92 and a concentration of 105% H_3_PO_4_. BGS also produced anhydrous phosphoric acids with specific gravity ranging between 1.90 and 1.92 to test the effect of acid water content on resultant isotope data. The acid is transferred to 500 ml glass bottles sealed with deformable plastic inserts and screw caps.

At UoL, approximately 500 ml of stock analytical grade 15 M H_3_PO_4_ is heated incrementally (25°C steps) under vacuum from 15°C to 165°C over a period of 6 days with evolved water removed cryogenically in a large diameter (5 cm) co‐axial trap held at −85°C. The procedure typically produces a solution with a specific gravity of between 1.93 and 1.95, and this is adjusted to 1.92 by addition of stock H_3_PO_4_. Specific gravity is measured using a commercially available hydrometer calibrated at 15.6°C. The solution is then heated at 140°C for 4 h (with continuous stirring) using a magnetic stirrer hot plate to ensure a compositionally homogenous product. The acid is finally transferred to 200 ml glass bottles sealed with deformable plastic inserts and screw caps, and stored in a vacuum desiccator.

At ETH,^33^ crystalline phosphoric acid is melted at 95°C and adjusted to the required composition by addition of P_4_O_10._ ^46^, ^49^ Notional concentrations of “100% H_3_PO_4_” and “104% H_3_PO_4_” are reported for acid used in sealed and open vessel methods, respectively, equivalent to specific gravities of approximately 1.86 and 1.91.

### 2.3 Preparation and analysis of CO_2_

The methods used to prepare and measure CO_2_ from siderite samples and calcite reference materials are very different between the BGS/UoL and ETH laboratories. Some additional experiments, designed to eliminate data differences due to the effects of individual laboratory reaction duration, anhydrous phosphoric acid concentration and siderite reaction rate, were conducted using the classical off‐line method at BGS and UoL. These experiments resulted in minor adjustments in BGS and UoL analytical protocols. Other than varying the specific variable under investigation, all other aspects of the method remain unchanged.

At BGS, the preparation of CO_2_ for the isotope analysis of siderite follows a version of the classical off‐line procedure described by McCrea^3^ and Swart et al.,^50^ and has been used since 1990. Sample carbonate, sufficient to yield approximately 130 μmol of CO_2_ (15 mg FeCO_3_), was weighed into 30 × 8 mm Durham tubes and reacted with 4 ml of anhydrous phosphoric acid of specific gravity (s.g.) ≈ 1.92 at 100.0 ± 0.2°C for 4 days in individual evacuated pear‐shaped flasks fitted with Viton® O‐ring sealed vacuum taps. A constant reaction temperature was maintained using a thermostatically controlled polyethylene glycol bath monitored with a PT100 digital thermometer. Product CO_2_ and H_2_O were subsequently separated under vacuum by cryogenic removal of water vapour at −80°C followed by transfer to gas collection vessels under liquid nitrogen. Any contaminant gases not condensed at −196°C were removed under vacuum prior to closure. The mass ratios (*m*/*z* 45/44, 46/44) of sample and standard CO_2_ were measured relative to a working comparison CO_2_ (reference gas) using a dual‐inlet, triple‐collector VG Optima gas‐source isotope ratio mass spectrometer. Results were converted to isotope delta (δ) values and corrected for ^17^O isobaric interferences on *m*/*z* 45.^51^, ^52^ Data were calibrated with respect to the international VPDB δ^13^C_VPDB_ and δ^18^O_VPDB_ reference scales.^31^ All δ^18^O values were adjusted for temperature‐dependent oxygen isotope fractionation associated with the carbonate‐phosphoric acid reaction using fractionation factors of 1.01025 for calcite at 25°C^53^ and 1.00881 for siderite at 100°C.^29^ For the purposes of within‐run calibration, each batch of siderite samples (typically 12 to 15 per batch) was accompanied by replicate preparation of CO_2_ from aliquots of a laboratory secondary calcite calibration material (MCS) with a carbon and oxygen isotope composition defined by measurement against NBS19 and normalized such that the δ^13^C and δ^18^O of NBS18 ≡ −5.01‰ and −23.01‰, respectively, on the VPDB scale.^31^ The MCS laboratory standard was reacted at 25.2 ± 0.1°C for 16 h and product CO_2_ recovered using procedures identical to those described for siderite samples.

At UoL, production of CO_2_ from siderite followed essentially identical gas preparation procedures to those used at BGS. The only departure from the BGS method weas sample carbonate, sufficient to yield approximately 50 μmol of CO_2_ (6 mg of FeCO_3_), was reacted with 1.5 ml of anhydrous phosphoric acid (s.g. ≈ 1.92) at 100.0 ± 0.2°C for 4 days. Constant reaction temperature was maintained using a thermostatically controlled glycerol‐water bath monitored with a PT100 digital thermometer. Product CO_2_ and H_2_O were subsequently separated under vacuum by cryogenic removal of water vapour at −90°C, followed by transfer to gas collection vessels under liquid nitrogen. Experiments to estimate the reaction rate of siderite with anhydrous phosphoric acid were undertaken at UoL by reacting 6‐mg splits (weighed to 0.001 mg) of a freshly crushed (<63 μm) cleavage fragment of the Ivigtut intercomparison material with 1.5 ml of acid at 100°C for between 0.5 and 96 h. Carbon dioxide yields were determined at constant volume using a linearising pressure transducer calibrated by measurement of CO_2_ produced from spectrographically pure CaCO_3_. The ambient temperature at measurement was recorded and all gas yields were adjusted to give pressure values at 298 K. Fractional yields were subsequently calculated relative to the total available CO_2_ for each sample. Measurement of sample and calibration CO_2_ followed identical principles to BGS using a dual inlet, triple‐collector VG SIRA 10 mass spectrometer. Resultant delta values were corrected for ^17^O isobaric interferences on *m*/*z* 45,^51^, ^52^ calibrated using mean values obtained from replicate within‐batch measurement of NBS19, normalized such that the mean δ^13^C and δ^18^O of replicates of NBS18 ≡ −5.01‰ and −23.01‰, respectively, on the VPDB scale,^31^ and δ^18^O values were adjusted using the same acid fractionation factors as BGS. Each batch of siderite samples (typically 8 to 12 per batch) was accompanied by preparation of CO_2_ (in triplicate) from aliquots of NBS19 and NBS18. Calibration materials were reacted at 25.0 ± 0.1°C for 16 h and product CO_2_ was recovered using procedures identical to those described for siderite.

Details of the sealed (on‐line) and open (off‐line) vessel gas preparation, measurement, and calibration methods used at ETH are reported in Fernandez et al.^33^ The sealed vessel (on‐line) method is based on the reaction of 200 μg of siderite in 12‐ml Exetainer® vials with 50 μl of “100%” (s.g. ≈ 1.86) anhydrous phosphoric acid at 70°C for up to 60 h using a GasBench® heating module to maintain reaction temperature. Reaction products were passed through a chromatography column and the purified CO_2_ transferred directly under a He stream to a mass spectrometer operating in continuous‐flow mode. Each batch of siderite samples was accompanied by the preparation of CO_2_ (under identical conditions) from two laboratory calcite materials for the purposes of within‐batch calibration. Open vessel (off‐line) procedures are based on the reaction of 15 mg of siderite with 1 ml of “104%” (s.g. ≈ 1.91) anhydrous phosphoric acid at 100°C in McCrea‐type (side‐arm) glass tubes for 3 h using a boiling water bath to maintain reaction temperature. Product CO_2_ was continuously extracted under liquid nitrogen and then purified initially by cryogenic (−80°C) trapping of H_2_O, followed by chromatographic separation to remove any organic contaminants. Product CO_2_ was flame sealed into Pyrex tubes before being transferred to a dual inlet mass spectrometer for mass ratio measurement. Each batch of samples was accompanied by the preparation of CO_2_ from four calcite materials for the purposes of within‐batch calibration.

## 3 RESULTS

### 3.1 Mineralogy and geochemistry

A summary of the mineralogical composition of each intercomparison material determined by XRD and a full tabulation of XRF and TC elemental analysis data is provided in Appendix S1. Quantitative estimates of the mineralogical composition of intercomparison materials were determined on the basis of geochemical (XRF, TC) data using a normative procedure in which key elements are assigned to specific minerals identified from XRD patterns (Table 3). Available information on the mineralogical and geochemical composition of ETH test siderites are reported in Fernandez et al.^33^ All materials were identified as pure siderite with varying, but elevated (X_FeCO3_ < 0.8) quantities of Mg, Ca, and Mn substitution.

**TABLE 3:** Mineralogical composition of siderite intercomparison materials determined by XRD

Material	Mineralogy (wt%)						
	Siderite	Quartz	Fluorite	Calcite	Phyllosilicates^b^	Apatite	Hematite
MR15423	55	37^a^	–	7	(C, M, K)	1	
MR17327	65	24	9	–	2 (?M)	–	
MR31556	85	5	–	–	10 (M)	–	
IVIGTUT	91	–	–	–		–	9^c^

^a^ Total quartz (plus phyllosilicate minerals).

^b^ Phyllosilicate (clay) minerals: C, chlorite; M, muscovite/illite; K, kaolinite.

^c^ Hematite (plus other iron oxides).

### 3.2 Carbon and oxygen isotopic composition

**FIGURE 1:**
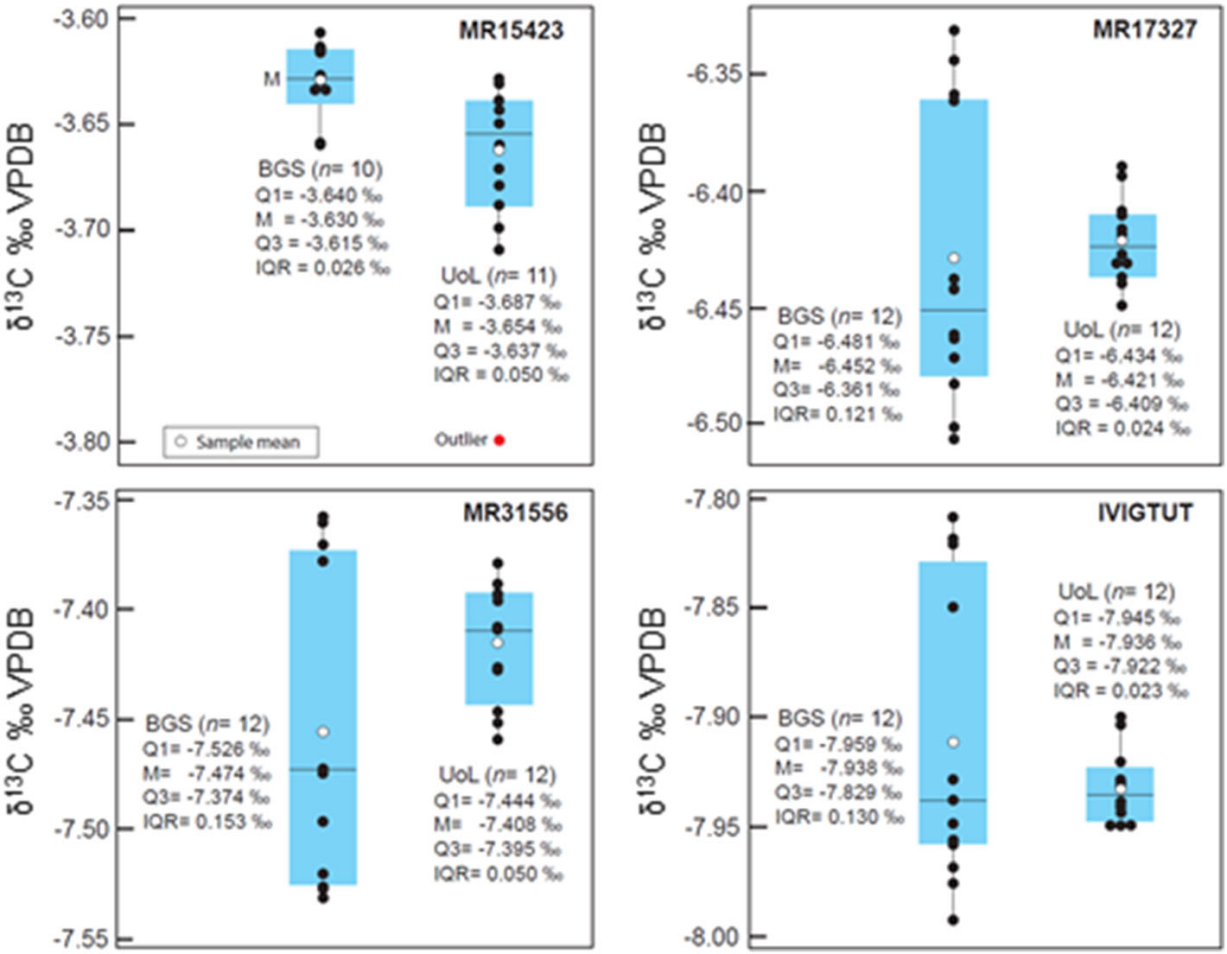
Comparison of δ^13^C values for siderite intercomparison materials summarised as box plots. BGS, British Geological Survey; UoL, University of Liverpool; Q1, first quartile; M, median; Q3, third quartile; IQR, interquartile range [Color figure can be viewed at wileyonlinelibrary.com]

**FIGURE 2:**
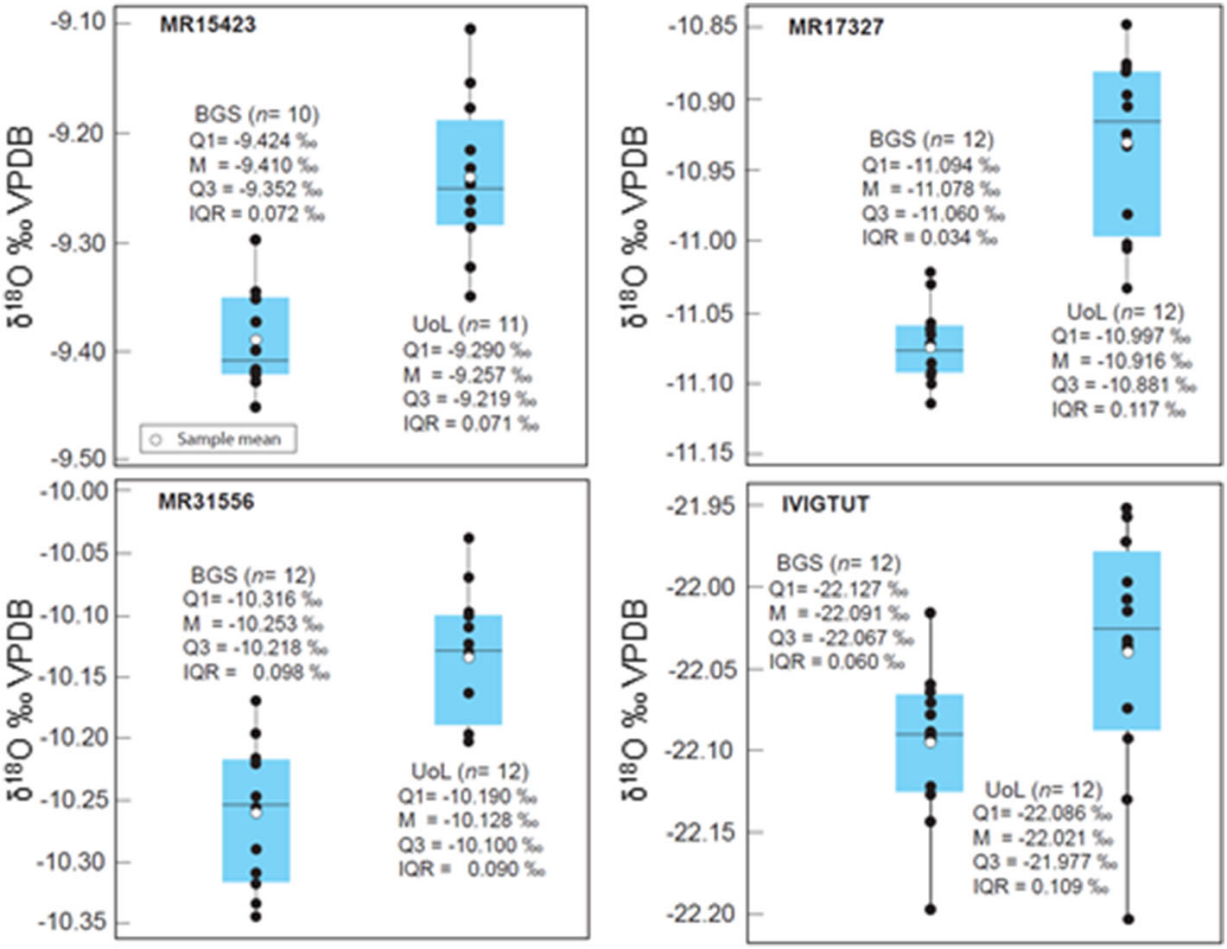
Comparison of δ^18^O values for siderite intercomparison materials summarised as box plots. BGS, British Geological Survey; UoL, University of Liverpool; Q1, first quartile; M, median; Q3, third quartile; IQR, interquartile range. The outlier in the UoL data set for MR15423 is not included in the statistical summary [Color figure can be viewed at wileyonlinelibrary.com]

Individual δ^13^C and δ^18^O data sets produced by the BGS and UoL laboratories are reported in Appendix S1. The siderite intercomparison data are illustrated as box plots in Figures 1 and 2, and a statistically reduced summary is provided in Tables 4 and 5. The δ^13^C and δ^18^O values fall within a relatively narrow range of values (δ^13^C = −3.6‰ to −7.9‰, δ^18^O = −9.3‰ to −22.1‰). Estimates of measurement repeatability based on individual sample population standard deviations are consistently <0.1‰.

**TABLE 4:** Summary of carbon isotope data for siderite intercomparison materials reacted off‐line at 100°C in sealed vessels

Material	Mean δ^13^C_VPDB_ ‰ (CI_95_)		SD (SE)		Difference (CI_95_)	*t*‐statistic (df)^a^	*p* value	*δ* ^b^
	BGS	UoL	BGS	UoL	BGS ‐ UoL			
MR15423	*n* = 10	*n* = 11	*n* = 10	*n* = 11				
	−3.630 (−3.643, −3.617)	−3.662 (−3.681, −3.644)	0.018 (0.006)	0.028 (0.008)	0.032 (0.011, 0.053)	3.17 (17)	0.006	0.035
MR17327	*n* = 12	*n* = 12	*n* = 12	n = 12				
	−6.431 (−6.471, −6.391)	−6.420 (−6.431, −6.410)	0.064 (0.018)	0.017 (0.005)	−0.011 (−0.052, 0.031)	−0.56 (12)	0.586	0.067
MR31556	*n* = 12	*n* = 12	*n* = 12	*n* = 12				
	−7.457 (−7.501, −7.412)	−7.416 (−7.434, −7.400)	0.070 (0.020)	0.028 (0.008)	−0.041 (−0.088, 0.006)	−1.87 (14)	0.082	0.057
IVIGTUT	*n* = 12	*n* = 12	*n* = 12	*n* = 12				
	−7.912 (−7.954, −7.869)	−7.932 (−7.943, −7.922)	0.067 (0.019)	0.015 (0.004)	0.020 (−0.023, 0.063)	1.01 (12)	0.330	0.052

Abbreviations: BGS, British Geological Survey; CI_95_, 95% confidence interval; DF, degrees of freedom; SD, standard deviation; SE, standard error; UoL, University of Liverpool.

^a^ Independent two‐tailed *t*‐test (*H*_0_: *μ*_BGS_ = *μ*_UoL_│*σ* ^2^_BGS_ ≠ *σ* ^2^_UoL_, *p* ≥ 0.05). ^b^Minimum detectable difference between true means (90% probability).

**TABLE 5:** Summary of oxygen isotope data for siderite intercomparison materials reacted off‐line at 100°C in sealed vessels (BGS: British Geological Survey, UoL: University of Liverpool)

Material	Mean δ^18^O_VPDB_ ‰ (CI_95_)		SD (SE)		Difference (CI_95_)	*t*‐statistic (df)^a^	*p* value	*δ* ^b^
	BGS	UoL	BGS	UoL	BGS ‐ UoL			
MR15423	*n* = 10	*n* = 11	*n* = 10	*n* = 11				
	−9.392 (−9.425, −9.358)	−9.255 (−9.294, −9.217)	0.047 (0.015)	0.057 (0.017)	−0.136 (−0.185, −0.088)	−5.94 (19)	<0.001	0.078
MR17327	*n* = 12	*n* = 12	*n* = 12	*n* = 12				
	−11.074 (−11.090, −11.060)	−10.931 (−10.970, −10.893)	0.028 (0.008)	0.060 (0.017)	−0.143 (−0.183, −0.104)	−7.48 (22)	<0.001	0.067
MR31556	*n* = 12	*n* = 12	*n* = 12	*n* = 12				
	−10.261 (−10.300, −10.220)	−10.134 (−10.168, −10.134)	0.057 (0.017)	0.053 (0.015)	−0.127 (−0.174, −0.080)	−5.63 (22)	<0.001	0.077
IVIGTUT	*n* = 12	*n* = 12	*n* = 12	*n* = 12				
	−22.097 (−22.130, −22.070)	−22.038 (−22.088, −21.993)	0.047 (0.014)	0.074 (0.021)	−0.060 (−0.112, −0.007)	−2.35 (22)	0.028	0.087

Abbreviations: BGS, British Geological Survey; CI_95_, 95% confidence interval; DF, degrees of freedom; SD, standard deviation; SE, standard error; UoL, University of Liverpool.

^a^ Independent two‐tailed *t*‐test (*H*_0_: *μ*_BGS_ = *μ*_UoL_│*σ* ^2^_BGS_ = *σ* ^2^_UoL_, *p* ≥ 0.05). ^b^Minimum detectable difference between true means (90% probability).

**FIGURE 3:**
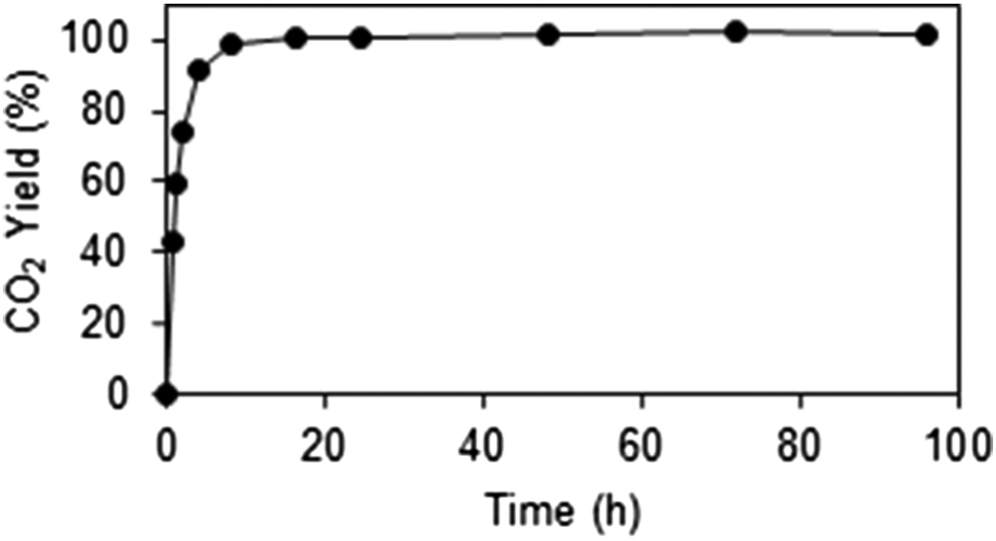
Fractional CO_2_ yield (moles CO_2_ recovered/moles CO_2_ siderite) as a function of reaction time (h) for Ivigtut siderite (<63 μm) reacted off‐line at 100°C in sealed vessels

The interlaboratory comparison of ETH test siderites was undertaken on a retrospective basis and the BGS and UoL measurements were completed approximately 12 months after the original ETH data had been published.^33^ In addition, restricted amounts (tens of milligrams) of the ETH test materials were available for analysis at BGS and UoL, allowing only a limited number (≤6) of repeat measurements to be undertaken. Individual δ^13^C and δ^18^O data sets produced by the BGS, UoL, and ETH laboratories are provided in Appendix S1. Only sample means, with δ^18^O values reported on the Vienna Standard Mean Ocean Water (VSMOW) scale, and standard deviations were available for ETH sealed‐vessel reaction at 70°C. The δ^18^O values were converted to the VPDB scale for comparison with other data sets. A statistically reduced summary is provided in Tables 6 and 7, and a visual comparison of sample means is illustrated in Figure 3. Values for δ^13^C and δ^18^O fall within a relatively narrow range of values (δ^13^C = −5.1‰ to −12.8‰, δ^18^O = −12‰ to −22‰). Classical procedures employed by BGS and UoL generate highly repeatable data (*σ* < 0.1‰) for all materials. By contrast ETH versions of the open (off‐line) and sealed (on‐line) vessel methods yield variable repeatability (*σ* = 0.014‰ to 0.328‰) as indicated by the data scatter illustrated in Fernandez et al.^33^ (Figure 2c and d therein). High variability between sample repeats (>0.3‰) observed in three ETH open‐vessel reactions at 100°C data sets is associated with a single extreme value (see Appendix S1). In two cases these extreme values were recognised as potential statistical outliers (*p* < 0.05) on the basis of a two‐tailed Grubb's test,^54^ but all available data were retained for comparison in this study.

**TABLE 6:** Summary of carbon isotope data for ETH test siderite materials

Material	Mean δ^13^C_VPDB_ ‰ (SD, SE)			
	BGS	UoL	ETH	ETH
	SV/100^a^	SV/100^a^	OV/100^b^	SV/70^c^
ES	*n* = 3	*n* = 6	*n* = 4	*n* = 11
	−5.141 (0.021, 0.012)	−5.243 (0.015, 0.006)	−5.117 (0.014, 0.007)	−5.122 (0.080, 0.024)
FOUR	*n* = 3	*n* = 6	*n* = 4	*n* = 9
	−7.083 (0.015, 0.009)	−7.193 (0.016, 0.006)	−6.954 (0.319, 0.159)	−7.021 (0.059, 0.020)
IG	*n* = 1	*n* = 3	*n* = 4	*n* = 12
	−8.02	−8.113 (0.006, 0.003)	−7.930 (0.189, 0.094)	−7.975 (0.062, 0.018)
LT	*n* = 3	*n* = 6	*n* = 4	*n* = 10
	−12.701 (0.014, 0.008)	−12.803 (0.031, 0.013)	−12.850 (0.328, 0.164)	−12.811 (0.081, 0.026)

Abbreviations: BGS, British Geological Survey; ETH, Eidgenössische Technische Hochschule; SD, standard deviation; SE, standard error; UoL, University of Liverpool.

^a^ Sealed vessel, off‐line, 100°C.

^b^ Open vessel, off‐line, 100°C.

^c^ Sealed vessel, on‐line, 70°C.

**TABLE 7:** Summary of oxygen isotope data for ETH test siderite materials (BGS: British Geological Survey, UoL: University of Liverpool, ETH: Eidgenössische Technische Hochschule)

Material	Mean δ^18^O_VPDB_ ‰ (SD, SE)			
	BGS	UoL	ETH	ETH
	SV/100^a^	SV/100^a^	OV/100^b^	SV/70^c^
ES	*n* = 3	*n* = 6	*n* = 4	*n* = 11
	−12.852 (0.009, 0.005)	−12.895 (0.055, 0.022)	−12.729 (0.063, 0.032)	−13.715 (0.154, 0.046)
FOUR	*n* = 3	*n* = 6	*n* = 4	*n* = 9
	−15.485 (0.104, 0.060)	−15.591 (0.050, 0.019)	−15.616 (0.114, 0.057)	−16.421 (0.107, 0.036)
IG	*n* = 1	*n* = 3	*n* = 4	*n* = 12
	−22.00	−22.173 (0.070, 0.041)	−22.173 (0.099, 0.049)	−23.070 (0.032, 0.009)
LT	*n* = 3	*n* = 6	*n* = 4	*n* = 10
	−15.467 (0.028, 0.016)	−15.565 (0.049, 0.020)	−15.621 (0.313, 0.157)	−16.453 (0.275, 0.087)

Abbreviations: BGS, British Geological Survey; ETH, Eidgenössische Technische Hochschule; SD, standard deviation; SE, standard error; UoL, University of Liverpool.

^a^ Sealed vessel, off‐line, 100°C.

^b^ Open vessel, off‐line, 100°C.

^c^ Sealed vessel, on‐line, 70°C.

### 3.3 Siderite reaction rate

The reaction rate of the Ivigtut intercomparison siderite at 100°C was investigated at UoL using a freshly crushed (<63 μm) cleavage fragment and the classical acid digestion method. The results are summarised in Figure 3. Rapid dissolution, in which approximately 90% of the total available sample CO_2_ was released, occurred within the first 4 h. The remaining 10% was released more slowly and the reaction appeared to be complete after 16 h.

### 3.4 Effect of phosphoric acid concentration on δ^13^C and δ^18^O

**FIGURE 4:**
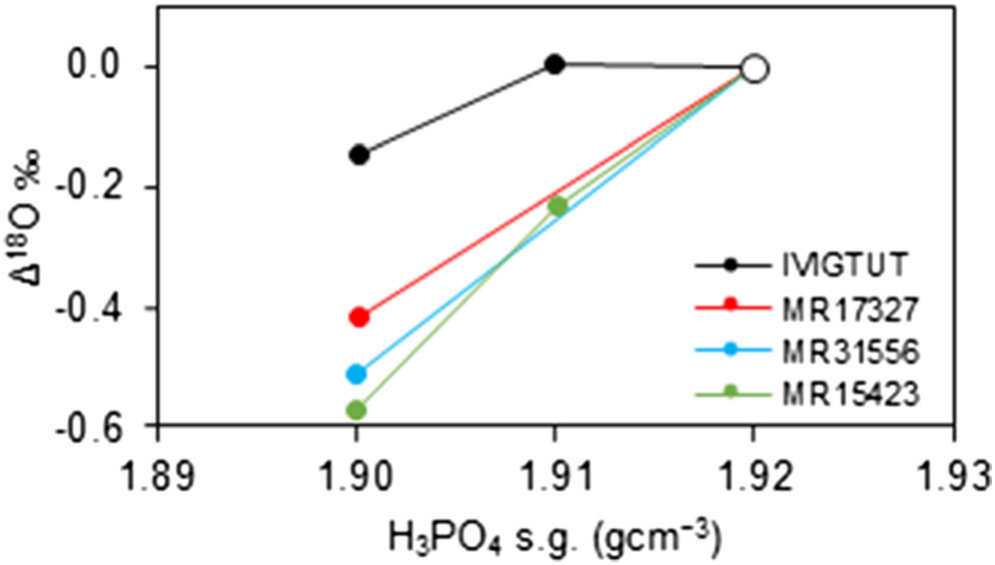
Measured δ^18^O values of liberated CO_2_ at a given specific gravity (s.g.) relative to the δ^18^O of liberated CO_2_ at a s.g. of 1.92 (Δ^18^O = δ^18^O_s.g. = *X*_ – δ^18^O_s.g. = 1.92_) as a function of the s.g. of anhydrous phosphoric acid measured for intercomparison siderite materials reacted off‐line at 100°C in sealed vessels [Color figure can be viewed at wileyonlinelibrary.com]

The effect of using anhydrous phosphoric acid of differing concentration, with specific gravities of between 1.90 and 1.92, on the isotope composition of CO_2_ produced from the intercomparison materials reacted for 96 h at 100°C was investigated at BGS using the classical acid digestion method. No significant change in δ^13^C of the liberated CO_2_ was recognised, but systematic increases in δ^18^O with increasing specific gravity were recorded (Figure 4).

### 3.5 Effect of reaction progress on δ^13^C and δ^18^O

**FIGURE 5:**
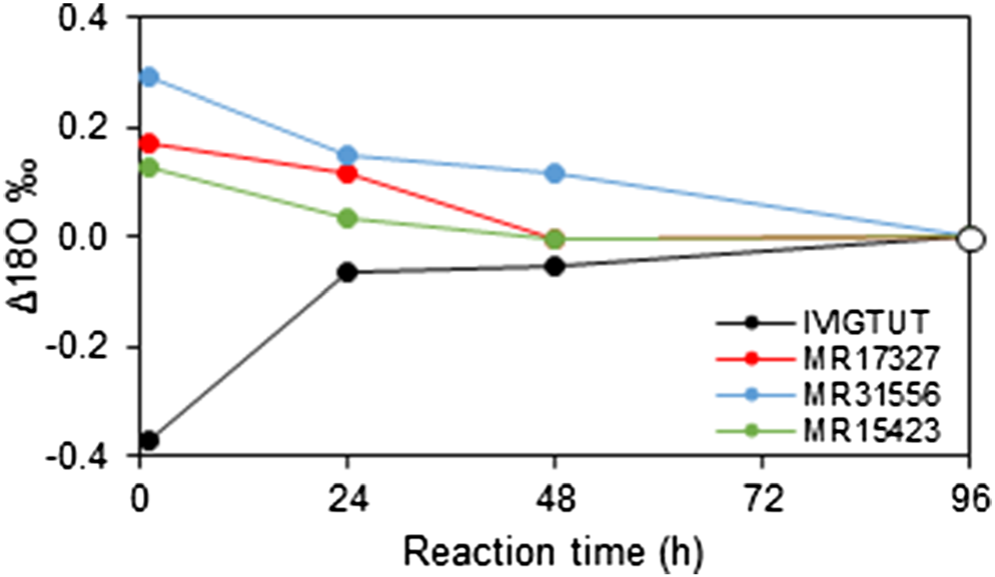
Measured δ^18^O values of liberated CO_2_ at a given reaction duration relative to the δ^18^O of liberated CO_2_ at a reaction duration of 96 h (Δ^18^O = δ^18^O_time = *X*_ – δ18O_time = 96h_) as a function of reaction duration for intercomparison siderite materials reacted off‐line at 100°C in sealed vessels [Color figure can be viewed at wileyonlinelibrary.com]

Experiments were undertaken at BGS using the classical acid digestion method to investigate the potential for change in the isotope composition of CO_2_ with reaction progress by reacting individual aliquots of each siderite material at 100°C for specified durations between 1 and 96 h. No significant differences were recorded in the δ^13^C with reaction time, but changes in δ^18^O were observed (Figure 5). Values for δ^18^O from the Ivigtut siderite were found to increase with reaction duration while values for other materials decreased over the same time intervals. In all cases, the data describe an asymptotic curve consistent with reaction systems evolving towards an apparent quasi‐steady state.

## 4 DISCUSSION

### 4.1 Siderite intercomparison materials

Differences (BGS − UoL) in mean δ^13^C values determined for all intercomparison materials are minimal (<±0.04‰) with no systematic offset and are considered to be within error (Figure 1 and Table 4). Two δ^13^C data sets from BGS (MR31556, Ivigtut) are characterised by positively skewed distributions and fail a Shapiro–Wilk normal distribution test (Appendix S1); we attribute this to the relatively small number of replicates (*n* ≤ 12) in sample populations. All δ^13^C data sets fail a Levene comparison of variance test (H_0_: *σ* ^2^_BGS_ = *σ* ^2^_UoL_) at the 0.05 level (Appendix S1), showing that the spread of data differs significantly between laboratories. Allowing for the small sample population and the tendency towards bimodality of the MR31556 and Ivigtut data sets, only MR15423 fails a two‐tailed comparison of sample means *t*‐test (H0: *μ*_BGS_ = *μ*_UoL_│*σ* ^2^_BGS_ ≠ *σ* ^2^_UoL_) at the 0.05 significance level (Table 4) and the mean δ^13^C values measured in each laboratory can be considered effectively identical.

In contrast to δ^13^C values, differences (BGS − UoL) in mean δ^18^O values exhibit a systematic offset of approximately −0.1‰. (Figure 2 and Table 5). All data sets pass a normal distribution test (Appendix S1) and only the data sets for MR17327 fail a comparison of variance test (H_0_: *σ* ^2^_BGS_ = *σ* ^2^_UoL_) at the 0.05 level (Appendix S1). However, all data sets fail a two‐tailed comparison of sample means *t*‐test (H0: *μ*_BGS_ = *μ*_UoL_│*σ* ^2^_BGS_ ≠ *σ* ^2^_UoL_) at the 0.05 significance level (Table 5), consistent with the occurrence of a small laboratory‐dependent measurement bias. Although both laboratories follow similar analytical protocols, and we tested specific aspects of the method, the cause of this bias has not been identified.

### 4.2 ETH test materials

As sample populations and variances of individual BGS, UoL, and ETH data sets differ widely, it is unrealistic to undertake a quantitative comparative statistical assessment. Consequently, only qualitative comparisons are discussed.

**FIGURE 6:**
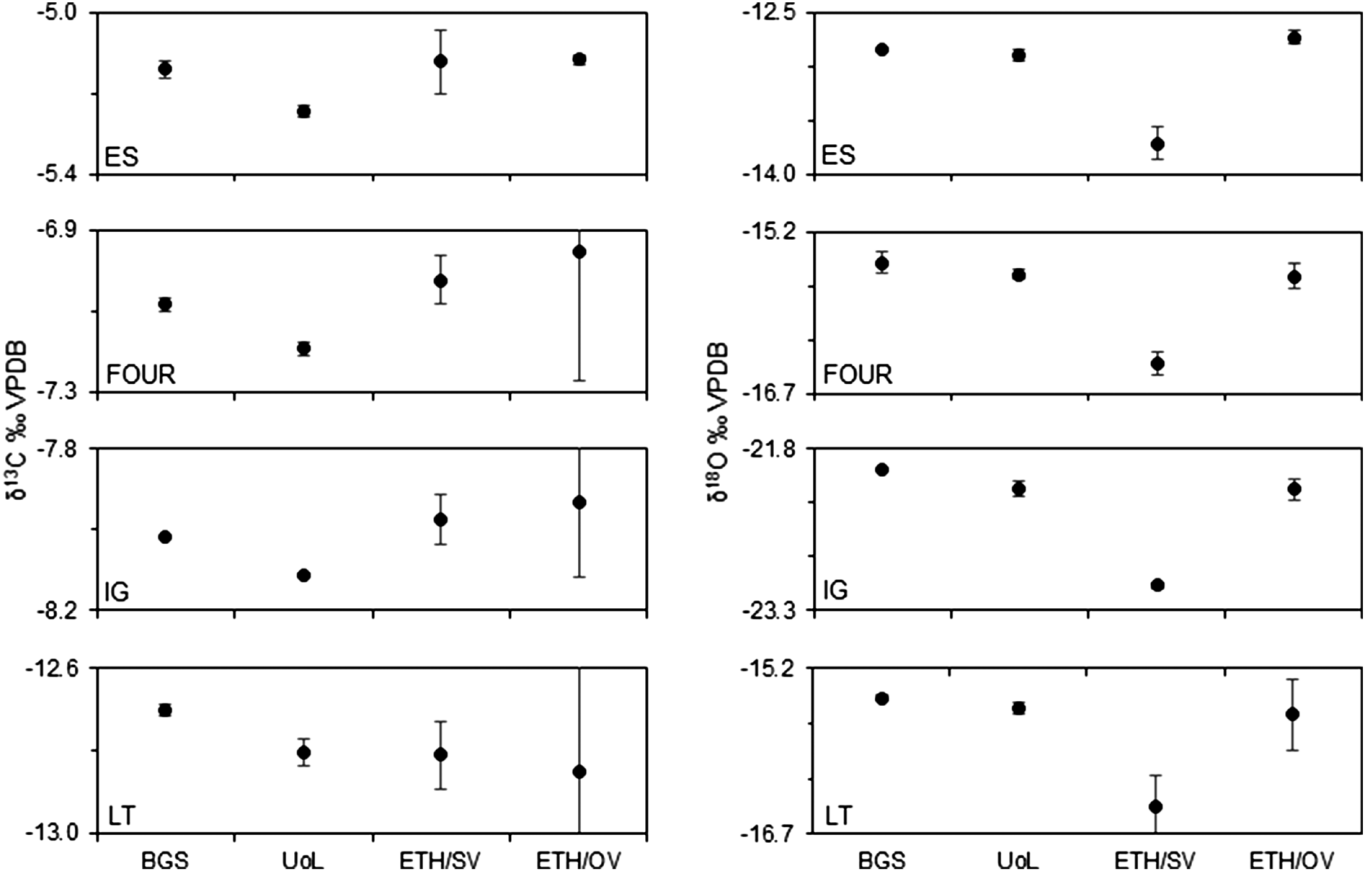
Comparison of mean δ^13^C and δ^18^O values determined for ETH test siderites at the British Geological Survey (BGS) and University of Liverpool (UoL) with those of Fernandez et al.^33^ measured at Eidgenössische Technische Hochschule (ETH). Reactions using sealed vessels were performed at 100°C off‐line for BGS/UoL and at 70°C on‐line for ETH, and for open vessels at 100°C off‐line for ETH

Mean δ^13^C values are in reasonable agreement and essentially independent of analytical method (*σ* ≤ 0.10‰). Maximum and minimum mean values for each material differ by <0.24‰ (Figure 6 and Table 6) and in at least two cases much of the discrepancy can be attributed to the inclusion of single extreme values in the ETH open‐vessel reactions at 100°C data statistics. Excluding these outliers, mean δ^13^C values differ by <0.18‰ (*σ* ≤ 0.08‰). Despite small sample populations and significant differences in analytical proficiency, measurement of δ^13^C on siderite can therefore be considered qualitatively robust and reproducible. Furthermore, the δ^13^C data sets yield a relatively consistent pattern of offset (Figure 6). Only the datum for the ETH LT sample open vessel (off‐line) reaction at 100°C deviates from this behaviour and this again is attributable to the inclusion of a single extreme measurement in the mean. The consistent pattern of response between these test materials implies that systematic laboratory‐specific measurement differences could effectively be eliminated by normalisation using suitable siderite reference materials. However, a suitable number of replicates should be used if employing the open‐vessel (off‐line) reaction at 100°C method given that outliers were present across three out of the four samples analysed by Fernandez et al.,^33^ resulting in higher associated analytical uncertainty (Table 6).

Mean δ^18^O values produced by open‐vessel (off‐line) reaction at 100°C agree in general with maximum and minimum mean values for each material differing by <0.16‰ (Figure 6 and Table 7). The pattern of offset between laboratories is consistent within uncertainties imposed by the lower measurement proficiency for the ETH open‐vessel (off‐line) reaction at 100°C data and suggests that interlaboratory offsets could be reduced by using approved reference materials. In comparison, mean δ^18^O values generated by the ETH sealed vessel (on‐line) reaction at 70°C are systematically lower by approximately 1‰ (Figure 6 and Table 7). This large disparity between δ^18^O values produced at 70°C on‐line and 100°C off‐line requires further investigation. Fernandez et al.^33^ discuss possible sources of measurement discrepancy, but obvious differences in reaction temperature and the concentration of anhydrous phosphoric acid provide the most likely explanation.

### 4.3 Factors contributing to siderite measurement inconsistency

#### 4.3.1 Sample preparation and storage

Pre‐analysis sample preparation, storage, and organic contamination removal procedures are all recognised factors potentially contributing to discrepancy in interlaboratory carbonate stable isotope measurement.^40^ In addition to issues identified for carbonates in general, we draw attention to two aspects that may be specific for samples of siderite.

Qi et al.^55^ have shown that the isotope composition of finely crushed carbonate materials is susceptible to modification during prolonged contact with humid air, even when stored in apparently tightly sealed screw‐cap vials. In addition, powdered carbonates that contain elevated concentrations of transition metals in a low oxidation state, such as siderite, may be particularly prone to progressive oxidation and loss of structural CO_2_ with time once crushed (Equation 4 and 5).

(4) $${\text{2FeCO}}_{3}+{\text{3H}}_{2}O+½O_{2}\to {\text{2CO}}_{2}+\text{2Fe}{\left(\mathrm{OH}\right)}_{3}$$

(5) $$\text{2Fe}{\left(\mathrm{OH}\right)}_{3}\to {\mathrm{Fe}}_{2}{\mathrm{CO}}_{3}+{\text{3H}}_{2}O$$

It is generally recommended that such materials are stored in a desiccator to minimise isotope exchange reactions mediated by water molecules absorbed on particle surfaces. To minimise these potential effects siderite samples should be analysed soon after crushing. For long‐term storage powders may need to be sealed in evacuated glass ampoules.

The use of methods to reduce or eliminate organic contaminants prior to isotope analysis is the subject of continued debate.^40^, ^56^, ^57^ We do not review the arguments here, but note that water‐based oxidising agents such as hydrogen peroxide and sodium hypochlorite have the potential to further modify the isotope composition of siderite by oxidation of ferrous iron. Lebeau et al.^16^ reported measurable shifts in the isotope composition of test siderites following treatment with both H_2_O_2_ and NaClO, and recommended the use of an oxygen plasma furnace as a viable alternative for removing organic contamination.

#### 4.3.2 Reaction rate

Rosenbaum and Sheppard^29^ documented exceptionally long reaction times (>>10^3^ h) for siderite under sealed vessel conditions at 25°C and proposed the use of elevated reaction temperatures (≥100°C) to compensate. Despite using a test siderite crushed to a particle size of <60 μm, reaction times >65 h at 100°C and >1 h at 150°C were apparently required for complete sample reaction. In contrast, broadly similar experiments reported by Carothers et al.^18^ and Fernandez et al.^33^ suggest that full reaction of siderite with phosphoric acid can occur on much shorter timescales.

Fernandez et al.^33^ noted that a complete reaction for the ETH test siderites was achieved within 50 h at 70°C for sealed vessels (80% yield after ~ 24 h gives isotope values within external analytical uncertainties of complete digestion) and 3 h at 100°C under open‐vessel conditions. These results are broadly consistent with our own sealed‐vessel experiments (Figures 3 and 5). Although no grain size distribution data are available for any of these yield experiments, tailing of CO_2_ yield curves is attributable to the extended time required to react a residual fraction of coarser particles and method‐dependent differences in the desorption rate of CO_2_ temporarily (or even permanently) absorbed within the phosphoric acid.

Differences in the rate of siderite dissolution at any specified reaction temperature are predicted to be dependent on a range of sample‐specific (particle size, sample mass, carbonate chemistry) and procedural (sealed versus open vessel, concentration of phosphoric acid) variables. Intuitively we would expect small quantities of finely powdered, highly substituted nonstoichiometric (*X*_FeCO3_ <<1) siderite to react significantly faster than large quantities of coarser, near‐stoichiometric (*X*_FeCO3_ ≈ 1) siderite. The quantitative effects of gas preparation variables are less evident. Open‐vessel procedures, where CO_2_ is continuously removed from the reaction system under liquid nitrogen, are likely to result in a faster reaction rate than sealed‐vessel procedures where all other variables remain constant. Similarly, “common acid bath” systems,^50^ where the sample and acid are continuously stirred, would also be expected to result in faster sample reaction rates than “static” procedures. Carbonate reaction rates may also depend on the specific concentration of anhydrous phosphoric acid, with small differences in water content potentially affecting acid viscosity, ion transport, rate of carbonate protonation, and CO_2_ desorption. The sensitivity of carbonate reaction rates to the concentration of anhydrous phosphoric acid requires further investigation.

#### 4.3.3 Effect of acid concentration on δ^18^O

Ghosh et al.^39^ and Wendeberg et al.^41^ document systematic changes in the δ^18^O value of CO_2_ produced from samples of calcite by reaction with anhydrous phosphoric acid of differing concentrations. Identical effects were also observed from H_3_PO_4_‐CO_2_ exchange experiments.^38^, ^43^ In all cases changes in isotope composition were attributed to differences in the availability, quantity, and δ^18^O value of H_2_O in the reaction system.

Similar changes are observed for CO_2_ prepared from siderite using anhydrous phosphoric acid of differing specific gravities in this study, but the gradients recorded from our experiments are significantly steeper than those reported previously.^39^, ^41^ A speculative explanation for this behaviour may be provided by the interaction of competing variables contributing to potential isotope exchange. These include relative differences in the water content of the acid, reaction rate, δ^18^O value of the acid and siderite, distribution of exchangeable oxygen between oxygen‐bearing species, system temperature, and experimental design.^38^, ^41^, ^43^ The much higher reaction temperature and extended reaction times used to prepare CO_2_ from siderite and the water content of anhydrous phosphoric acid may be key factors affecting both the rate and magnitude of the isotope exchange effect.^41^, ^43^ Defliese et al.,^42^ for example, record an exponential increase in the vapour pressure of H_2_O over anhydrous phosphoric acid in sealed‐vessel systems, with pressure increasing sharply above 50°C, and highlight the possible consequences for isotope exchange with CO_2_.

Although we did not determine either the δ^18^O value of anhydrous phosphoric acid or the water vapour in the sealed vessels used in our experiments (cf. Wendeberg et al.^41^ and Swart et al.^43^) we note that the anhydrous phosphoric acid concentration‐dependent gradients (Figure 4) are identical for siderite materials with similar δ^18^O and depart markedly from the relationship defined by the Ivigtut siderite (characterised by a much lower δ^18^O). This is taken to imply that the effective δ^18^O of the Ivigtut siderite (Table 5) is likely to be much closer to the δ^18^O value of the acid than other intercomparison siderites. The apparent magnitude of this effect is noteworthy because it appears to indicate that at high reaction temperatures in the region of 100°C the measured δ^18^O of CO_2_ is particularly sensitive to the water content of the phosphoric acid and the relative difference between the effective δ^18^O of the acid and sample carbonate. This observation requires further quantitative investigation.

#### 4.3.4 Effect of reaction progress on δ^18^O

Changes in the δ^18^O value of CO_2_ with reaction progress have been reported for calcite, dolomite, and siderite.^26^, ^33^, ^39^, ^58^ Walters et al.^26^ argued that observed shifts in isotope composition were related to the effects of carbonate particle size and the rate of diffusion of reaction products from particle surfaces. Alternatively, Fernandez et al.^33^ emphasised the effects of isotope exchange between competing oxygen‐bearing species in the FeCO_3_–CO_2_–H_2_O–H_3_PO_4_ reaction system.

In this study, the δ^18^O of CO_2_ released from aliquots of Ivigtut siderite increased with reaction duration and for other materials the δ^18^O of CO_2_ decreased with time (Figure 5). As intercomparison siderite MR15423 contains a significant quantity of calcite (≈7 wt%), we are unable to assign any significance to the behaviour of this material. However, the fact that a similar response is observed in two other materials suggests that the isotope effect may be real.

A time‐sensitive effect related to the rate of carbonate reaction, relative difference in the δ^18^O of siderite and phosphoric acid, and the temperature‐dependent fractionation and rate of isotope exchange between CO_2_, H_2_O and phosphate species at the time of CO_2_ extraction provides a plausible explanation for observed changes. During the initial stages of reaction, a dynamic, isotope disequilibrium state should persist until the siderite reaction has terminated. However, it is also possible that the entire oxygen isotope exchange system will continue to evolve towards a steady state, dependent on the kinetics of individual exchange reactions, over time periods well beyond the point at which the carbonate reaction has actually ceased.^29^, ^38^

The direction and magnitude of these variables on the evolving δ^18^O of CO_2_ will ultimately be dependent on the method of CO_2_ production and extraction, with different outcomes arising from differences in system variables including the isotope composition of reactants, particle size of sample carbonate, concentration of phosphoric acid, reaction temperature, and design of the gas preparation apparatus. A full mechanistic description of the isotope systematics is beyond the scope of this study, but a qualitative treatment of potential oxygen isotope exchange vectors is presented by Swart et al.^43^ The outcome of our experiments implies that the δ^18^O value of MR17327 and MR31556 is higher than the δ^18^O of phosphoric acid, but lower for the Ivigtut siderite.

With reaction progress, any isotope fractionations related to the rate of carbonate bond breaking and CO_2_–H_2_O–H_3_PO_4_ exchange should tend towards a steady state and all reactions involved in the preparation of analyte CO_2_ should ideally attain equilibrium prior to gas recovery. Consequently, sufficient time is required to both react all sample carbonate and to allow the isotope system to reach at least quasi‐equilibrium. From our experiments we note that the δ^18^O of product CO_2_ continues to evolve (>24 h) after the apparent siderite reaction has reached completion (<24 h). We are currently unable to account for this disparity, but suggest that oxygen isotope equilibration of the CO_2_–H_2_O–H_3_PO_4_ system continues after the siderite reaction has reached completion (cf. Swaert et al.^43^). Although contrary to the observations of Rosenbaum and Sheppard,^29^ our empirical data suggest that at 100°C the chosen reaction duration should be applied consistently to minimise any potential time‐sensitive compositional drift.

#### 4.3.5 Siderite acid fraction factor

Three published studies report estimates for the temperature‐dependent fractionation of oxygen isotopes during the production of CO_2_ by reaction of siderite with anhydrous phosphoric acid between 25°C and 150°C.^18^, ^29^, ^33^ Fernandez et al.^33^ combined these data sets to produce an equation describing the relationship across the entire temperature range for all sealed‐vessel siderite digestions:

(6) $$1000\ln\left(\alpha_{{\mathrm{CO}}_{2}\;\text{siderite}}\right)=4.06\left(\frac{1000}{T}\right)-1.93$$

where *T* is temperature in Kelvin.

Although this approach provides a relationship with broad utility, integration of fractionation factors derived using differing methodologies are unlikely to be internally consistent. Fernandez et al.^33^ suggest uncertainties in the estimate of 1000 ln ($\alpha_{{\mathrm{CO}}_{2}-\text{siderite}}$) equivalent to ±0.5‰ based on their compilation and emphasise the need for further validation. Apparent method‐dependent differences in $\alpha_{{\mathrm{CO}}_{2}-\text{siderite}}$ reported by Fernandez et al.^33^ between the sealed (on‐line) and open (off‐line) vessel methods are not supported by oxygen isotope data produced from the classical sealed (off‐line) vessel method used in this study. Data from the BGS and UoL sealed‐vessel reactions at 100°C and ETH open‐vessel reactions at 100°C agree in general and imply that the observed isotopic difference between ETH versions of sealed‐ and open‐vessel procedures may be causally linked to differences in reaction temperature and the concentration of anhydrous phosphoric acid. The evident disparity in δ^18^O values determined for siderite reaction at 70°C and 100°C deserves further investigation.

In addition to uncertainties associated with estimates of $\alpha_{{\mathrm{CO}}_{2}-\text{siderite}}$, the potential consequences of differences in siderite chemistry on the oxygen isotope fractionation effect are poorly defined. Rosenbaum and Sheppard^29^ derived an empirical equation describing an apparent relationship between $\alpha_{{\mathrm{CO}}_{2}-\text{siderite}}$ (at 100°C) and the chemical composition of rhombohedral carbonates based on simple ideal mixing of end‐members:

(7) $$1000\ln\left(\alpha_{{\mathrm{CO}}_{2}\;\text{siderite}/100{}^{\circ}C}\right)=9.29X_{{\text{MgCO}}_{3}}+8.94X_{{\text{CaCO}}_{3}}+8.77X_{{\text{FeCO}}_{3}}$$

where *X*_*i*_ is the mole fraction of component *i* in a rhombohedral carbonate.

Justification for this behaviour is provided by Guo et al.,^27^ who compared empirical and theoretical (cluster model) fractionation factors for end‐member carbonates and inferred a linear, although not precise, relationship between $\alpha_{{\mathrm{CO}}_{2}-\text{siderite}}$ and the reciprocal of cation radius (1/*r*). The coefficients derived by Rosenbaum and Sheppard^29^ agree closely with an experimentally determined fractionation factor for magnesite (1000 ln*α* = 9.19) reported by Sharma et al.,^59^ but disagree markedly with estimates of the calcite fractionation factor (1000 ln*α* ≈ 7.90) compiled by Kim et al.^31^

Given that the siderite lattice can accommodate significant quantities of Mg, Ca, and Mn, the specific chemical composition may need to be considered when comparing the δ^18^O of siderites with differing chemistries. The compositional dependency of $\alpha_{{\mathrm{CO}}_{2}-\text{siderite}}$ at 100°C defined by Rosenbaum and Sheppard^29^ requires further corroboration, but the overall effects are likely to be minor (<0.5‰) when compared with other sources of analytical uncertainty, even for siderites with significant Mg or Ca substitution.

### 4.4 Siderite reference materials

The use of globally available, matrix‐specific reference materials to improve the reproducibility of isotope data is well established.^36^, ^60^ Currently there is a lack of carbonate‐specific reference materials and only calcite is adequately catered for. Modification of the classical acid digestion method to accommodate slowly reacting carbonate minerals and the development of automated gas preparation techniques has furthered the requirement for siderite reference materials to minimise method‐dependent bias in measurement data produced by individual laboratories. The comparative data presented in this study emphasise the need for this approach when measuring δ^18^O. Application of the principle of identical treatment via agreed reference siderites would also remove the need to apply poorly defined and potentially laboratory‐specific oxygen isotope fractionation factors, although effects related to differences in siderite chemistry would remain.

The location and preparation of suitable materials (in sufficient quantities), value assignment, quality control/quality assurance, and storage and distribution/administrative costs all present significant challenges for the production of acceptable reference materials. Value assignment may prove to be particularly problematic if large method‐dependent oxygen isotope bias is realised in other laboratories. Conformity to ISO standards as recommended by the International Atomic Energy Agency,^60^ cost implications, and the level of demand for siderite reference materials mean that acceptable materials are unlikely to become available without concerted effort from the carbonate stable isotope analytical community. However, until suitable reference materials become available the isotopic measurement of carbonate minerals, other than calcite, must be subject to method‐dependent uncertainties that propagate through to the quantitative interpretation of isotopic data in terms of natural geochemical processes.

### 4.5 Recommendations for isotope analysis of siderite

We make the following suggestions for improving the repeatability and reproducibility of δ^13^C and δ^18^O values. Given the proliferation of automated gas preparation systems (based on multiple combinations of gas preparation conditions) and differing mass spectrometry currently in use it is impossible to establish a standard protocol for routine analysis. The challenge of producing consistent data sets can only be resolved practically by the global availability of siderite reference materials and the reliance on identical treatment as a basis for reducing method‐dependent contributions to local measurement bias.

- To minimise reaction times a sample particle size ≤200 μm is necessary. However, fine‐grained siderite powders may be subject to rapid oxidation in contact with moist air. Storage in a desiccator is a minimum requirement and analysis soon after crushing is recommended. The use of water‐based oxidising agents to remove organic contamination should be avoided.
- While the effects of differing acid preparation methods on carbonate measurement are undetermined, siderite δ^18^O values are sensitive to differences in the concentration of phosphoric acid. Use of anhydrous phosphoric acid with a narrow specific gravity range of 1.92 (H_3_PO_4_ ≈ 105%) and 1.93 (H_3_PO_4_ ≈ 106%) is suggested to balance the need to reduce reaction time and the amount of H_2_O in the reaction system.
- No attempt has been made to assess the effects of differences in sample mass and phosphoric acid volume. Maintaining a consistent, laboratory‐defined ratio between these variables is advisable. It is noted that mixing larger quantities of fine‐grained siderite powder with smaller volumes of phosphoric acid can lead to solidification issues potentially arresting the reaction before completion and trapping a portion of the liberated CO_2_.
- Elevated reaction temperatures are required for efficient sample processing. A temperature of 100°C offers a compromise between reaction rate limitations and the potential negative effects from the high vapour pressure of H_2_O in the sealed‐vessel headspace.
- The integrated effects of temperature, particle size, mineral chemistry, and concentration of phosphoric acid on siderite reaction rate are poorly defined. We recommend a minimum reaction time of 24 h at 100°C for sealed‐vessel procedures. The use of a consistent reaction time should minimise effects related to possible time–temperature‐sensitive oxygen isotope equilibration between CO_2_‐H_2_O‐H_3_PO_4_.
- When using a generic $\alpha_{{\mathrm{CO}}_{2}-\text{siderite}}$ and discounting impurities, this will affect the accuracy of the data for specific samples but not the precision achieved on the data. In the absence of rigorous determination of $\alpha_{{\mathrm{CO}}_{2}-\text{siderite}}$, we recommend the continuing use of the fractionation factors of Rosenbaum and Sheppard.^29^ Extrapolation to lower temperatures (<<100°C) should be avoided. Possible method‐dependent differences of $\alpha_{{\mathrm{CO}}_{2}-\text{siderite}}$ between sealed‐ and open‐vessel procedures reported by Fernandez et al.^33^ have not been confirmed in this study and the use of method‐specific fractionation factors requires validation.
- The application of the principle of identical treatment, using published oxygen isotope fractionation factors ($\alpha_{{\mathrm{CO}}_{2}-\text{carbonate}}$) for siderite samples and calcite calibration materials, which may not be internally consistent at elevated reaction temperatures, provides the only available procedure for automated determination of the isotope composition of siderite but may result in poorer measurement reproducibility.

## 5 CONCLUSIONS

Determination of δ^13^C values in samples of siderite using differing gas preparation and measurement protocols yields both repeatable and reproducible data at an acceptable level of reproducibility (*σ* < 0.1‰, on mean values). Conversely, δ^18^O values appear to be far more sensitive to method‐dependent effects, leading potentially to much poorer measurement reproducibility (up to ~1‰ offset between different techniques). The development of globally available reference materials is required to improve measurement reproducibility. Normalisation to a defined siderite‐specific scale (relative to VPDB), based on reliable reference materials, should mitigate local, method‐dependent bias across the range of manual and automated gas preparation and measurement procedures currently in use. However, poorly defined uncertainties associated with existing estimates of $\alpha_{{\mathrm{CO}}_{2}-\text{siderite}}$ and the effects of siderite chemistry on $\alpha_{{\mathrm{CO}}_{2}-\text{siderite}}$ remain.

## COMPETING INTEREST STATEMENT

The authors declare no competing interests.

### PEER REVIEW

The peer review history for this article is available at https://publons.com/publon/10.1002/rcm.9456.

## Supporting information


**Appendix S1.** Supporting Information

## Data Availability

The data that supports the findings of this study are available in the supplementary material of this article.
